# QuickStats

**Published:** 2014-06-06

**Authors:** FROM THE NATIONAL CENTER FOR HEALTH STATISTICS

**Figure:**
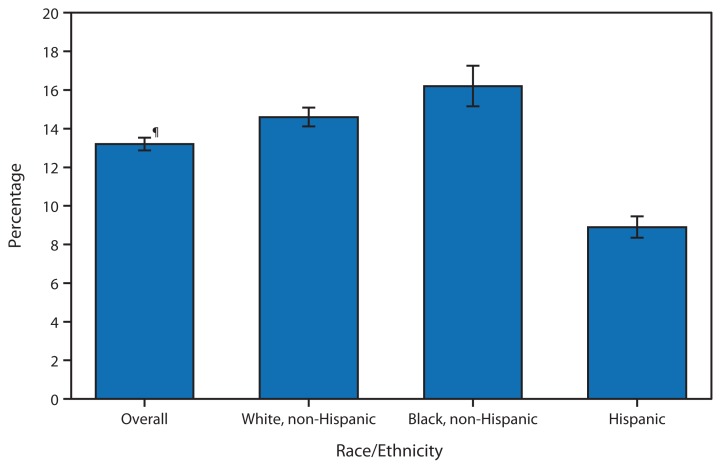
Percentage of Children Aged <18 Years with a Health Problem for Which They Have Taken Prescription Medication Regularly for ≥3 Months,* by Race/Ethnicity^†^ — National Health Interview Survey, United States, 2012^§^ ^*^ Based on a survey question that asked respondents, “Does [your child] now have a problem for which [he/she] has regularly taken prescription medication for at least 3 months?” Unknowns were not included in the denominators when calculating percentages. ^†^ Children of Hispanic ethnicity might be of any race or combination of races. Non-Hispanic children are not of Hispanic ethnicity, regardless of race. ^§^ Estimates are age-adjusted using the projected 2000 U.S. population as the standard population and using age groups 0–4 years, 5–11 years, and 12–17 years. ^¶^ 95% confidence interval.

In 2012, overall, 13% of children aged <18 years had a health problem for which prescription medication had been taken regularly for ≥3 months. Non-Hispanic white children (15%) and non-Hispanic black children (16%) were more likely to have taken a regular medication for a health problem for ≥3 months than Hispanic children (9%).

**Source:** Bloom B, Jones LI, Freeman G. Summary health statistics for U.S. children: National Health Interview Survey, 2012. Vital Health Stat 2013;10(258).

**Reported by:** Gulnur Freeman, MPA, grs3@cdc.gov, 301-458-4085; Bobbie Bloom, MPA; Lindsey Jones, MPH.

